# The AI implementation gap in trauma radiography: standalone *versus* discretionary AI-integrated fracture detection

**DOI:** 10.1186/s41747-026-00763-6

**Published:** 2026-06-16

**Authors:** Anna Gurabi, Sirus S. Hosseini-Begtary, Peter Hegedus, Andor Viktor Gal, Pal Maurovich-Horvat, Nikolett Marton

**Affiliations:** 1 Department of Radiology, Clinic for Medical Imaging, Semmelweis University, Budapest, Hungary; 2 Department of Diagnostic and Interventional Radiology, Heidelberg University Hospital, Heidelberg, Germany; 3 Department of Neuroradiology, Clinic for Medical Imaging, Semmelweis University, Budapest, Hungary

**Keywords:** Artificial intelligence, Deep learning, Emergency medicine, Fractures (bone), Radiography

## Abstract

**Objective:**

In emergency trauma care, artificial intelligence (AI) may aid fracture detection on radiographs, potentially reducing radiologists’ workload. We evaluated the role of deep learning-based decision-support software in the reporting of trauma cases.

**Materials and methods:**

We retrospectively analyzed 2317 trauma radiographs acquired at a single center: 1,174 images obtained from November 1 to 16, 2023, without access to the AI tool during reporting, and 1,143 images from February 1 to 13, 2024, with discretionary use of the AI output during reporting. The AI software output was compared with final radiology reports, with ground truth established by a musculoskeletal radiologist with 9 years’ experience. Accuracy, sensitivity, specificity, positive predictive value (PPV), and negative predictive value (NPV) were calculated at both the fracture and patient levels.

**Results:**

The dataset included 1,914 patients with 1,188 acute fractures (621 in November, 567 in February). At the fracture level, standalone AI achieved 90.7% accuracy, 87.8% sensitivity, 94.0% specificity, 94.3% PPV, and 87.2% NPV in November, 94.1%, 93.5%, 94.6%, 94.5%, and 93.6% in February, respectively. Non-AI-assisted radiologists reached 92.4%, 89.0%, 96.2%, 96.3%, and 88.7%, AI-assisted radiologists 93.4%, 90.0%, 96.7%, 96.4%, and 90.7%, respectively. At the patient level, AI’s overall performance reached up to 96.5% accuracy and 95.6% sensitivity. Discrepancies between AI and radiologists occurred in 326 cases, often related to anatomical variants such as accessory ossicles.

**Conclusion:**

Standalone AI demonstrated near-expert accuracy and sensitivity in fracture detection at both fracture and patient levels. PPV increased with AI support, indicating more accurate detection of actual fractures.

**Relevance statement:**

By examining discretionary real-world use of AI in trauma radiography, this study shows that clinical benefit is not guaranteed by algorithmic performance alone, as optional AI integration does not consistently improve radiologist sensitivity, underscoring a critical implementation gap in practice.

**Key Points:**

- Standalone AI achieves near-expert fracture detection performance in trauma radiography.
- Discretionary AI use does not consistently improve radiologist sensitivity.
- AI use reduces discrepancies, suggesting improved diagnostic consistency.
- Clinical benefit of AI depends on real-world implementation strategy.

**Graphical Abstract:**

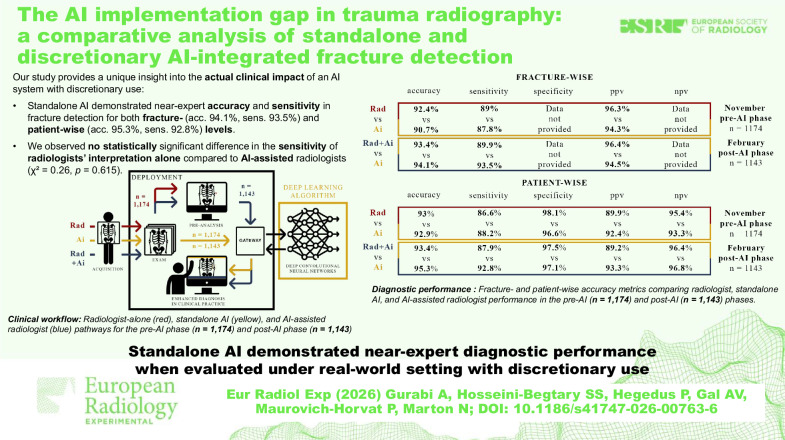

## Background

An increasing patient workload in the health care system is observable, which is linked, among other things, to the aging population and the increase in long-term chronic diseases and comorbidities [1, 2]. Many health associations worldwide have identified overcrowding in emergency departments (EDs) as a public health problem [3]. In the USA, 20% of all ED visits resulted in hospital admission [4]. A common diagnostic error in the ED is the missed detection of fractures on radiographs, which can range between 2% and 9% [5, 6]. The rate of non-detection or misinterpretation of fractures is higher at night, when a consultant radiologist is not always reachable in all hospitals [7]. Nevertheless, bone fractures are the most common injury type and represent the largest fraction of traumatological care in adults [8].

Traditional x-ray-based fracture diagnosis can often be time-consuming, depending on the experience of the diagnosing physician, and prone to human error (*e.g*., satisfaction of search, resulting in the possibility of missing multiple fractures present [9]), especially when it comes to more complex cases or hardly detectable fractures [10].

Artificial intelligence (AI) systems can help to reduce the workload on radiologists [7]. Machine learning is a subcategory of AI that involves algorithms allowing computers to learn from data and recognize patterns, then make decisions based on the information. They are able to generalize from given specific examples to make precise decisions or predictions on new and previously unseen data [7, 11]. Deep learning is a subset of machine learning, referring to the use of multiple layers of neural networks to access a higher level of knowledge in reaction to any input information, thus making it more efficient for image analysis [6, 12, 13].

Currently, there are already more than 200 European Conformity‒CE-marked commercial AI solutions in the field of radiology [14, 15]. BoneView software is one of the most extensively verified products with outstanding sensitivity and specificity [16]. Studies with the product have also proven a reduction in x-ray reading time [17]. Further benefits of using AI may include improved accuracy and efficiency, enhanced diagnostic performance, accessibility, and cost-effectiveness [18].

While numerous studies have validated the technical performance of AI in controlled settings, there remains a critical gap in understanding its clinical utility when integrated into real-world workflows without a mandatory usage protocol. The translation of algorithmic accuracy into tangible diagnostic benefit is not guaranteed and depends heavily on human-computer interaction.

Recent studies have increasingly evaluated AI performance under real-world or clinically realistic conditions. Prospective and crossover reader studies have shown that AI assistance may reduce reading time and increase diagnostic confidence, while often failing to produce consistent improvements in diagnostic accuracy or sensitivity for fracture detection when compared with unaided radiologists [19]. Comparative studies of commercially available fracture detection algorithms, including BoneView, have demonstrated high standalone performance but also highlighted variability across algorithms and challenges in detecting subtle abnormalities [20]. A recent systematic review focusing on BoneView reported heterogeneous effects on sensitivity and specificity across readers and study designs, emphasizing methodological limitations and the need for further real-world evaluations [21]. Moreover, real-life observational studies comparing AI-aided radiologists, emergency physicians, and standalone AI systems have shown that human performance remains superior in routine clinical settings, underscoring the complexity of translating algorithmic accuracy into clinical benefit [22]. Despite these advances, most existing studies rely on protocolized AI usage, controlled reader designs, or prospective awareness of AI support, leaving uncertainty regarding the diagnostic impact of discretionary AI use embedded into routine emergency radiology workflows.

Therefore, the primary objective of this study was to compare the diagnostic performance of a standalone AI algorithm with that of radiologists working both with and without discretionary AI assistance in a routine emergency trauma environment. We hypothesized that discretionary AI use may not lead to significant improvements in diagnostic accuracy, highlighting the importance of implementation strategy.

## Methods

### Study design

In our retrospective, single-center study, the radiographic records of patients and their clinical reports written by radiologists were analyzed. The study population consisted of two distinct, non-overlapping cohorts collected during separate time frames to evaluate different diagnostic workflows.

The first period (pre-AI implementation; 1–16 November 2023) included examinations interpreted by radiologists without access to the AI tool during clinical reporting. For these cases, standalone AI analysis was performed retrospectively by applying the AI software to the archived radiographs after routine reporting was completed. The second period (post-AI implementation; 1–13 February 2024) evaluated an integrated workflow where radiologists had optional real-time access to the AI output during the reporting process at their own discretion. All radiologists involved in reporting during this period had equal access to the AI tool. However, its use was not mandatory and was left entirely to the individual radiologist’s discretion.

**Fig. 1:**
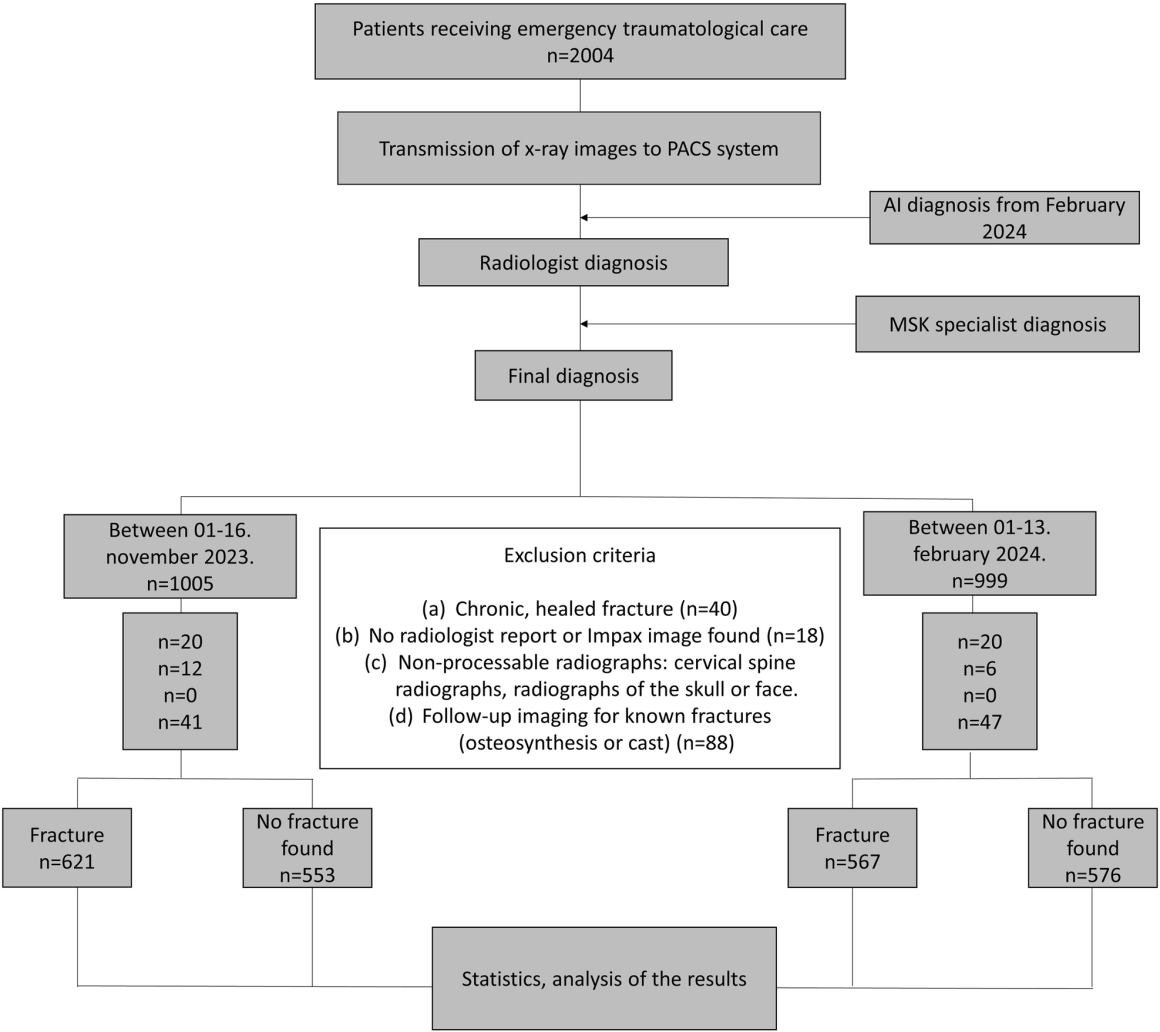
AI-assisted clinical workflow. The flowchart shows the evaluation of the performance of AI and radiologists in identifying fractures in patients arriving at the emergency department (ED) for trauma care. In 2004 patients were admitted from the Emergency Department. The radiographs were successfully transmitted to the PACS system, and then they were interpreted by radiology residents and verified by a radiology specialist. By February 2024, radiologists had the option of subjecting images to AI for diagnosis. The ground truth diagnoses were stated by a musculoskeletal radiologist with 9 years of experience. Patients were divided into two cohorts for comparative analysis of diagnostic performance across different timeframes: the first cohort included patients presenting between November 1 and November 16, 2023 (*n* = 1,005), and the second comprised patients seen between February 1 and February 13, 2024 (*n* = 999). Radiographs of chronic, healed fractures (*n* = 40); no radiologist’s interpretation or radiographic images were available (*n* = 18); x-rays of the cervical spine, skull, and face, as the AI software used in our study is not able to analyze these regions; and radiographs taken to follow up already detected fractures (*n* = 88) were excluded. After exclusion criteria were applied, in the November 2023 cohort (*n* = 1,005), a total of 621 cases were diagnosed with a fracture, while 553 cases were determined to have no fracture. In the February 2024 cohort (*n* = 999), 567 cases were identified as having a fracture, whereas 576 cases were classified as having no fracture. After that, a statistical analysis was performed, comparing the performance of radiologists and the AI system. AI, Artificial intelligence

Patient’s age in the first cohort was 54.2 ± 21.8 (mean ± standard deviation), in the second cohort 54.2 ± 22.5; patient’s sex was 50.4% female, 49.6% male, and 50.1% female, 49.9% male, respectively. All included radiographs were required to be of sufficient technical quality (free from significant motion artifacts, severe exposure issues, or positioning errors resulting in incomplete anatomical coverage). Image quality was verified retrospectively by an expert musculoskeletal radiologist with 9 years of experience. Our exclusion criteria were as described below (Fig. 1). Examinations with inadequate image quality were excluded from the analysis. Our exclusion criteria included: radiographs of chronic, healed fractures; x-rays of the cervical spine, skull, and face, as the AI software used in our study is not able to analyze these regions; radiographs taken to follow up already detected fractures; and no radiologist’s interpretation or radiographic images were available. Cases with osteosynthesis and casts were excluded as well. The radiographs were labeled by anatomical regions (chest, spine, upper limb, and lower limb), with each region subdivided into bone levels.

A radiologist and an AI diagnosis were performed for each included radiograph. During February 2024, radiologists could choose to submit radiographs for AI analysis during report generation, whereas for the November cohort, AI results were generated retrospectively and were not available to radiologists at the time of interpretation. All patients were admitted under emergency trauma care, where they were examined by a clinician, and if the injury was considered suspicious for fracture, a conventional radiograph of the affected region was ordered.

The radiographs were performed by radiographers, followed by immediate access to the images in the picture archiving and communication system (PACS) system (Phönix-PACS GmbH). They were pre-interpreted by radiology residents and then verified by radiology specialists. The radiologists’ diagnoses were classified into positive, doubtful, and negative categories (for comparison with the AI software output). After the clinical introduction of AI, the radiographs were transmitted directly from the PACS system to the AI fracture detection software (BoneView version 2.6.0, Gleamer), and the AI results were returned to the PACS for review during reporting. Reference (ground truth) diagnoses were determined without clinical information by a musculoskeletal radiologist with 9 years of experience. Figure 1 shows the AI-assisted clinical workflow. The images of emergency trauma radiographs generated during these time intervals were included in our analysis. The AI fracture detection system can generate three outcomes: “Positive”, “Doubt”, or “Negative”. Each of these diagnoses is associated with a threshold value. If “Positive”, this value is greater than or equal to 90%, “Doubt” is between 50-90%, and “Negative” is below 50%.

### Statistical analysis

Data collection and analysis were performed using Excel.

For statistical analysis, all suspicious “Doubt” findings were considered positive, allowing for maximizing the number of fractures detected.

The following performance metrics were calculated to evaluate the diagnostic performance:

$${{\mathrm{Accuracy}}}=\frac{{{\mathrm{True}}}\,{{\mathrm{positive}}}+{{\mathrm{True}}}\,{{\mathrm{negative}}}}{{{\mathrm{True}}}\,{{\mathrm{positive}}}+{{\mathrm{False}}}\,{{\mathrm{positive}}}+{{\mathrm{True}}}\,{{\mathrm{negative}}}+{{\mathrm{False}}}\,{{\mathrm{negative}}}}$$

$${{\mathrm{Sensitivity}}}=\frac{{{\mathrm{True}}}\,{{\mathrm{positive}}}}{{{\mathrm{True}}}\,{{\mathrm{positive}}}+{{\mathrm{False}}}\,{{\mathrm{negative}}}}$$

$${{\mathrm{Specificity}}}=\frac{{{\mathrm{True}}}\,{{\mathrm{negative}}}}{{{\mathrm{True}}}\,{{\mathrm{negative}}}+{{\mathrm{False}}}\,{{\mathrm{positive}}}}$$

$${{\mathrm{Positive}}}\,{{\mathrm{predictive}}}\,{{\mathrm{value}}}\,({{\mathrm{PPV}}})=\frac{{{\mathrm{True}}}\,{{\mathrm{positive}}}}{{{\mathrm{True}}}\,{{\mathrm{positive}}}+{{\mathrm{False}}}\,{{\mathrm{positive}}}}$$

$${{\mathrm{Negative}}}\,{{\mathrm{predictive}}}\,{{\mathrm{value}}}\,({{\mathrm{NPV}}})=\frac{{{\mathrm{True}}}\,{{\mathrm{negatives}}}}{{{\mathrm{true}}}\,{{\mathrm{negatives}}}+{{\mathrm{False}}}\,{{\mathrm{negatives}}}}$$

Two analyses were performed, one at the patient level, where a positive result was considered if the patient had at least one fracture, providing a clinically relevant overview of diagnostic outcomes, and one at the fracture level, where each fracture was evaluated as an independent case. Confidence intervals were calculated based on the standard binomial (Wald) method directly on proportions.

$${{\mathrm{Confidence}}}\,{{\mathrm{interval}}}\,({{\mathrm{CI}}})=p\pm z\sqrt{\frac{p(1-p)}{n}}$$

Where *p* is the observed sensitivity, *n* is the total number of individuals with the condition (true positive + false negative), and *z* is the 1.96 multiplier for a 95% interval.

The χ^2^ and McNemar tests were used to determine statistical significance, where a *p*-value < 0.05 was considered significant. Although an *a priori* sample size calculation was not performed, a post-hoc analysis based on McNemar’s test confirmed that the sample size was sufficient to detect a relevant 2% difference in sensitivity between the AI and the radiologist (requiring 391 positive cases *versus* > 400 included).

### AI declaration

The authors used ChatGPT (GPT-5, OpenAI; accessed September 2025) to assist with language editing, grammar correction, and summarization of text during manuscript preparation. The authors reviewed, verified, and edited all AI-assisted content to ensure its accuracy and take full responsibility for the integrity and originality of the final manuscript.

## Results

### Standalone AI and pre-AI radiologists’ performance

A total of 955 patients with trauma-related musculoskeletal radiographic examinations were retrospectively evaluated in the first cohort, from which 429 patients’ 621 fractures were confirmed (Table 1). Resulting in a total of 1,174 cases, where negative examinations were counted as one case each, and positive examinations were counted according to the number of confirmed fractures.

**Table 1:** Patient and examination demographics

Characteristic	Pre-AI cohort (Nov 2023)	AI-Assisted cohort (Feb 2024)	Total
Number of patients	955	959	1,914
Mean age ± standard deviation (years)	54.2 ± 21.8	54.2 ± 22.5	54.2 ± 22.1
Sex (female, *n* (%))	481 (50.4%)	478 (50.1%)	959 (50.1%)
Number of examinations	1,174	1,143	2,317
Number of confirmed fractures	621	567	1,188

*AI* Artificial intelligence

A total of 182 discrepancies between the AI and radiologist assessments were identified, corresponding to a discrepancy rate of 15.6%. Fracture-wise, the AI system correctly interpreted 1065 cases, yielding an accuracy of 90.7%, sensitivity of 87.8%, specificity of 94.0%, PPV of 94.3%, and NPV of 87.3%. The reporting radiologist achieved 1,085 correct interpretations, with an accuracy of 92.4%, sensitivity of 89.0%, specificity of 96.2%, PPV of 96.3%, and NPV of 88.7%. There were 63 cases in which only the AI detected a fracture missed by the radiologist, and 71 cases where only the radiologist made the correct detection (Table 2). At the patient level, AI performance reached an accuracy of 93.8%, sensitivity of 90.4%, specificity of 96.6%, PPV of 95.6%, and NPV of 92.5%. Radiologist performance at this level was similarly strong, with an accuracy of 95.9%, sensitivity of 93.2%, specificity of 98.1%, PPV of 97.56%, and NPV of 94.7% (Table 3).

**Table 2:** Diagnostic performance at the fracture level

Metric (%, 95% CI)	Standalone AI (Nov 2023)	Radiologists (Nov 2023)	Standalone AI (Feb 2024)	AI-assisted radiologists (Feb 2024)
Accuracy	90.7% [89.1%–92.4%]	92.4% [90.9–93.9]	94.1% [92.7‒95.4%]	93.4% [91.9-94.8]
Sensitivity	87.8% [85.2–90.3]	89.1% [86.6–91.5]	93.5% [91.4–95.5]	89.9% [87.5–92.4]
Specificity	94.0% [92.0–96.0]	96.2% [94.6–97.8]	94.6% [92.8–96.5]	96.7% [95.2–98.2]
Positive predictive value	94.3% [92.4–96.2]	96.3% [94.8–97.9]	94.5% [92.6–96.5]	96.4% [94.8–98.0]
Negative predictive value	87.3% [84.5–89.9]	88.7% [86.1–91.2]	93.6% [91.7–95.6]	90.7% [88.4–93.0]

*AI* Artificial intelligence, *CI* Confidence interval

**Table 3:** Diagnostic performance at the patient level

Metric (%, 95% CI)	Standalone AI (Nov 2023)	Radiologist (Nov 2023)	Standalone AI (Feb 2024)		AI-Assisted radiologists (Feb 2024)
Accuracy	93.8% [92.3–95.4]	95.9% [94.7–97.2]	96.5% [95.3–97.6]		96.5% [95.3–97.6]
Sensitivity	90.4% [87.7–93.2]	93.2% [90.9–95.6]	95.6% [93.6–97.6]		95.1% [93.0–97.2]
Specificity	96.6% [95.0–98.1]	98.1% [96.9–99.3]		97.1% [95.7–98.5]	97.5% [96.2–98.8]
Positive predictive value	95.6% [93.6–97.6]	97.6% [96.1–99.1]		96.0% [94.1–97.9]	96.5% [94.7–98.3]
Negative predictive value	92.5% [90.3–94.7]	94.7% [92.8–96.6]		96.4% [95.3–98.2]	96.4% [94.9–98.0]

Diagnostic performance, considering a case positive if ≥ 1 fracture was present

*AI* Artificial intelligence, *CI* Confidence interval

### Radiologists’ performance with discretionary AI support

The second cohort comprised 959 patients with trauma-related musculoskeletal radiographic examinations, from which 567 fractures of 405 patients were confirmed. Similar to the previous cohort, negative examinations were counted as one case each, while positive examinations were counted according to the number of fractures, resulting in a total of 1,143 cases. Discrepancies between the AI and radiologist + AI interpretations occurred in 144 cases, yielding a discrepancy rate of 12.6%. As in the previous cohort, diagnostic performance was assessed at both the fracture- and patient-wise levels. Fracture-wise, the AI alone correctly interpreted 1,075 cases, corresponding to an accuracy of 94.1%, a sensitivity of 93.5%, a specificity of 94.6%, a PPV of 94.5%, and an NPV of 93.6%. Radiologists using AI support achieved slightly fewer correct interpretations (1,067), with an accuracy of 93.4%, a sensitivity of 90.0%, and an NPV of 90.7%, but demonstrated a higher specificity of 96.7% and a PPV of 96.4%. Notably, the AI detected 57 fractures that were missed by the radiologist, while the radiologist identified 37 fractures that were missed by the AI. Patient-wise, the AI system achieved an accuracy of 96.5%, sensitivity of 95.6%, specificity of 97.1%, PPV of 96.0%, and NPV of 96.8%. While the AI-assisted radiologist interpretation reached an accuracy of 96.5%, a sensitivity of 95.1%, a specificity of 97.5%, a PPV of 96.5%, and an NPV of 96.4%.

### Comparative diagnostic performance analysis

To assess the statistical significance of observed differences in diagnostic performance, both χ² and McNemar’s exact tests were applied at the fracture-wise and patient-wise levels. At the fracture-wise level, χ² analysis comparing the standalone AI sensitivity across the two reporting settings revealed a statistically significant improvement in the AI’s detection rate when integrated into the clinical workflow (χ² = 11.24, *p* = 0.0008). Similarly, AI accuracy also significantly increased between the two cohorts (χ² = 9.13, *p* = 0.0025), suggesting improved robustness of the algorithm in identifying fractures. In contrast, the sensitivity of human interpretation alone compared to AI-assisted radiologists showed no statistically significant difference (χ² = 0.25, *p* = 0.615), indicating that the addition of AI support did not universally enhance human sensitivity. Interestingly, the standalone AI system and radiologist performed similarly on the first cohort in terms of both sensitivity (*p* = 0.546) and accuracy (*p* = 0.159), highlighting the non-inferiority of the algorithm. This was further supported by McNemar’s exact test comparing the sensitivity of the AI-assisted radiologist *versus* standalone AI on the second cohort (*p* = 0.049). These results collectively suggest that the AI system alone may match or even outperform radiologists under certain conditions, although AI-assisted human interpretation may introduce variability depending on how the AI output is incorporated (Table 4).

**Table 4:** Statistical comparison of key performance metrics

Comparison	Metric	Test	Test statistic	*p*-value
Between-cohort comparisons (independent groups)				
Standalone AI (Nov) *versus* standalone AI (Feb)	Sensitivity (fracture)	χ²	11.24	0.0008
Standalone AI (Nov) *versus* standalone AI (Feb)	Accuracy (fracture)	χ²	9.13	0.0025
Radiologists (Nov) *versus* AI-assisted radiologists (Feb)	Sensitivity (fracture)	χ²	0.25	0.615
Within-cohort comparisons (paired groups)				
Standalone AI (Feb) *versus* AI-assisted radiologists (Feb)	Sensitivity (fracture)	McNemar	37.0	0.049
Standalone AI (Feb) *versus* AI-assisted radiologists (Feb)	Accuracy (patient)	McNemar	34.0	1.000
Standalone AI (Feb) *versus* AI-assisted radiologists (Feb)	Sensitivity (patient)	McNemar	18.0	0.871

*AI* Artificial intelligence, *NA* Not available

Patient-level diagnostic performance revealed a statistically significant improvement in sensitivity for standalone AI between the two cohorts (*χ²* = 8.28, *p* = 0.004), as well as a significant increase in overall diagnostic accuracy (*χ²* = 7.17, *p* = 0.007). These findings indicate that the AI system became more effective in identifying fractured patients when used in routine clinical conditions. However, when comparing the patient-level sensitivity of radiologists with and without AI support, no significant difference was observed (*χ²* = 1.25, *p* = 0.264). Likewise, AI-assisted radiologists showed no statistically significant improvement in accuracy over standalone radiologists (*χ²* = 0.38, *p* = 0.539), suggesting that the benefit of AI assistance in this setting may be limited or inconsistently applied in real-world usage. McNemar’s exact test provided further insight into sensitivity and accuracy differentials within the same patient population. For patient-wise analysis, the difference between AI and AI-assisted radiologists did not approach statistical significance for either sensitivity (*p* = 0.871) or accuracy (*p* = 1.000). These results suggest that the AI system operating independently may be as consistent as radiologists selectively incorporating AI outputs.

### Analysis of diagnostic discrepancies

**Fig. 2:**
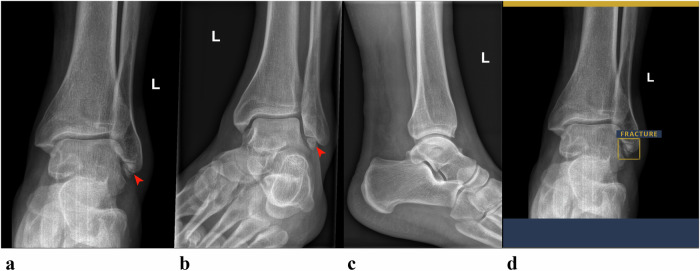
Anatomical variant. A 44-year-old male patient presented with localized swelling and pressure sensitivity over the lateral malleolus following blunt trauma after kicking a stone. Physical examination revealed intact skin and egg-sized swelling around the left ankle. The radiographic evaluation included anteroposterior, mortise, and lateral views. The imaging showed a well-corticated bone fragment adjacent to the lateral malleolus, consistent with an *os subfibulare*, a known anatomical variant, rather than an acute fracture. **a**–**c** Standard anteroposterior, mortise, and lateral ankle radiographs showing the *os subfibulare* (red arrows). **d** AI system analysis erroneously highlights the ossicle as a fracture (yellow box), resulting in a false-positive detection. Expert adjudication confirmed the presence of a non-pathological ossicle and ruled out an acute fracture, highlighting the importance of contextual interpretation in cases involving anatomical variants. This case illustrates a common diagnostic pitfall in automated systems, which may misinterpret anatomical variants as pathology in the absence of contextual clinical correlation. AI, Artificial intelligence. Source: Medical Imaging Centre, Semmelweis University, Budapest

**Fig. 3:**
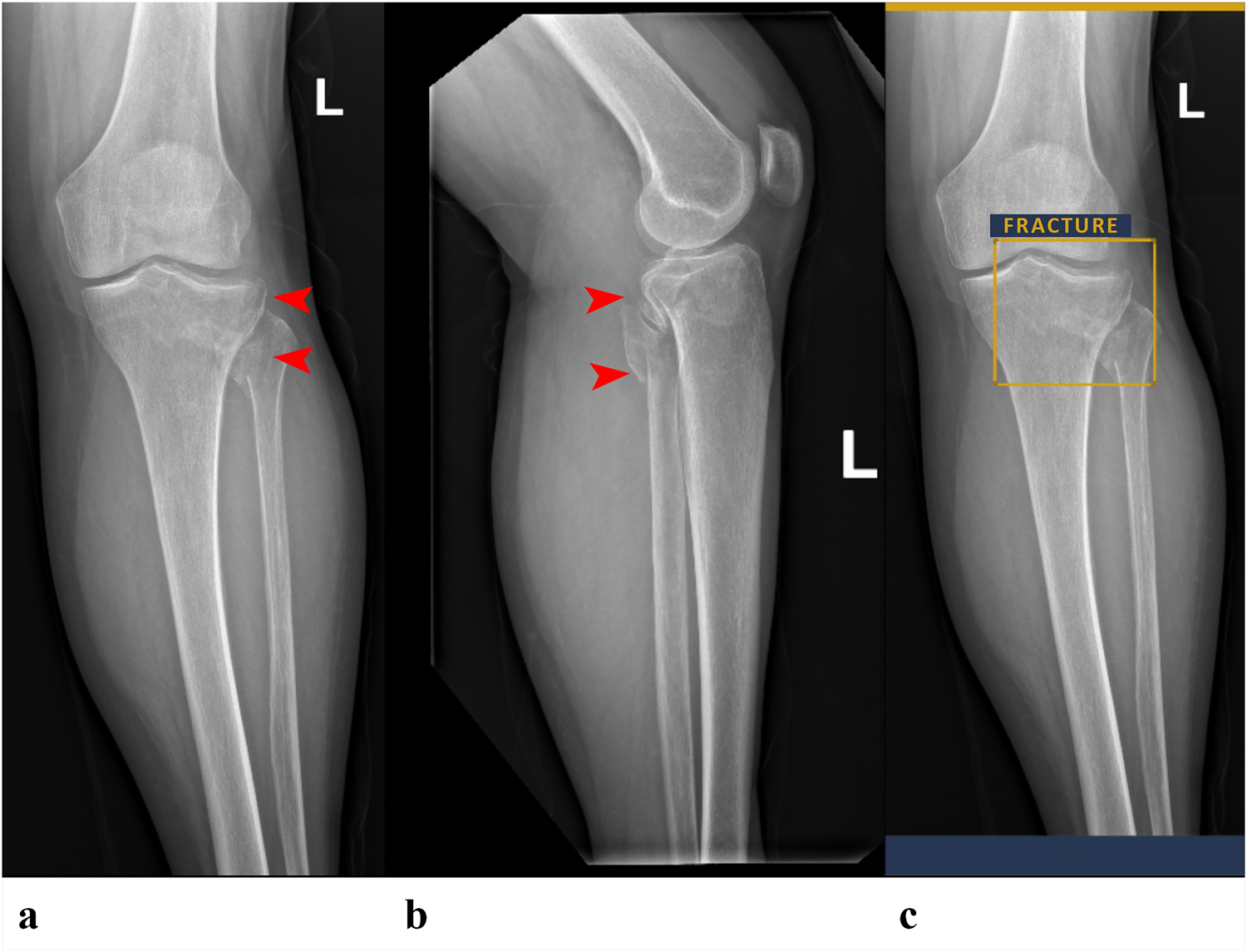
Misinterpretation of fracture number. The 70-year-old female patient presented with radiating knee pain after falling from a bicycle. Clinical examination revealed pain on palpation and preserved peroneal function. Knee extension was intact. The Radiographic evaluation of the left knee revealed two distinct fractures in the proximal tibia and in the proximal fibula. **a** Anteroposterior x-ray image showing clear fracture lines in both the proximal tibia and fibula (red arrows). **b** Lateral view confirming the fracture morphology and localization in both bones (red arrows). **c** AI analysis highlights the affected area with a bounding box (yellow box) but fails to differentiate the two anatomically distinct fracture sites. Instead, a single box is rendered that encompasses both fractures. Expert adjudication confirmed the presence of two separate fractures. The AI correctly flagged the injured area but misclassified the fracture count, identifying two fractures as a single lesion. This case illustrates a limitation of current AI systems in segmenting closely located but distinct fractures. AI, Artificial intelligence. Source: Medical Imaging Centre, Semmelweis University, Budapest

**Fig. 4:**
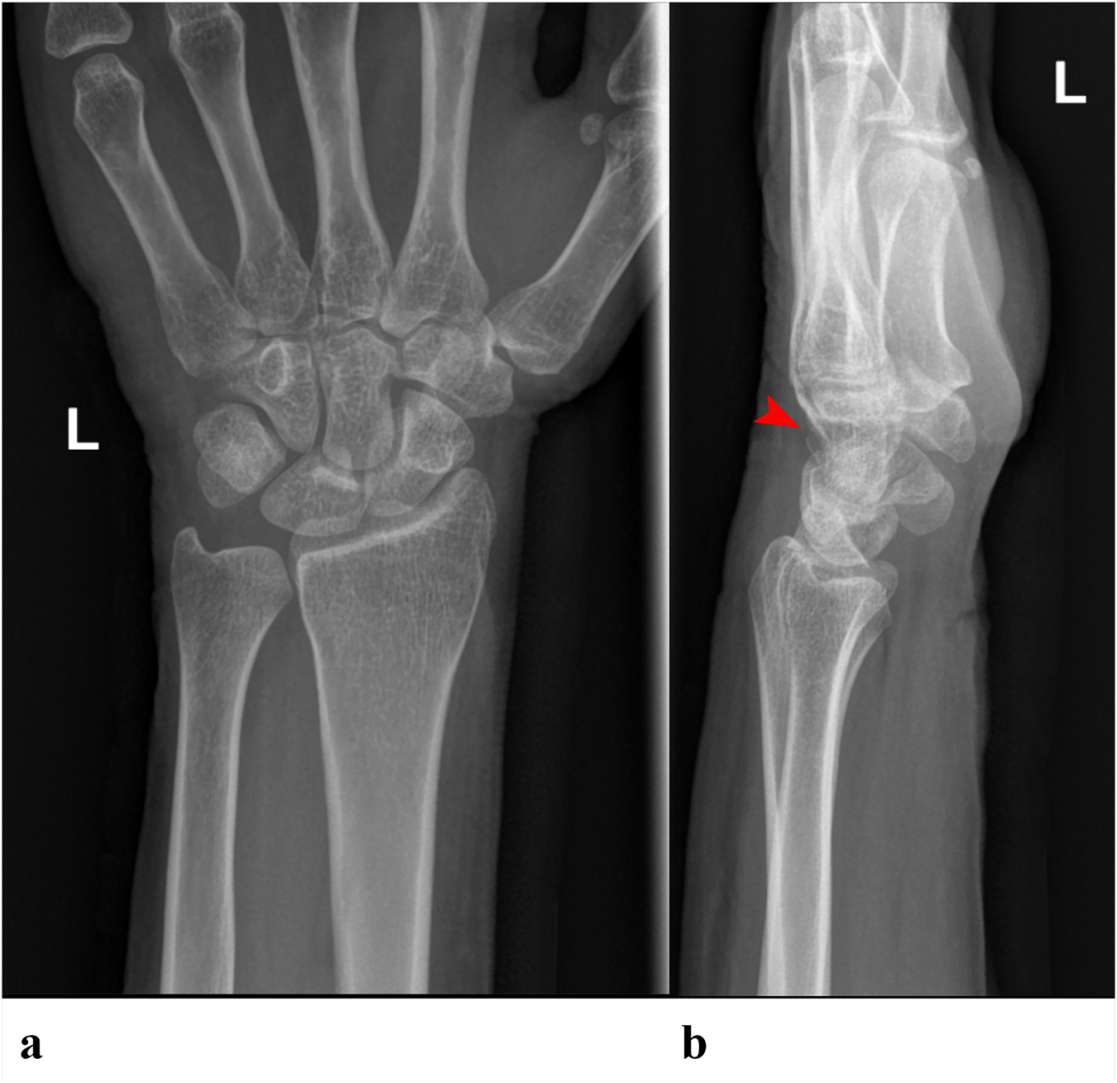
Missed avulsion fracture. The 41-year-old female patient presented to the outpatient clinic the following day after injuring herself at home with glass, arriving with persistent localized pain in the left wrist. The radiographic examination included anteroposterior and lateral views of the left wrist. Despite clear clinical symptoms, neither the AI system nor the radiologist identified the subtle avulsion fracture of the os triquetrum. **a** Anteroposterior x-ray image of the left wrist, where the fracture is not reliably appreciable. **b** Lateral x-ray image of the wrist demonstrating a subtle dorsal fracture line of the os triquetrum (red arrow). Expert adjudication later confirmed a small cortical fracture at the dorsal aspect of the os triquetrum, a known site for subtle trauma-related injuries that are often missed in standard reads. This case underscores a diagnostic blind spot shared by both AI and human interpretation in detecting small carpal bone fractures, highlighting the ongoing need for radiologist vigilance even in AI-supported workflows. AI, Artificial intelligence. Source: Medical Imaging Centre, Semmelweis University, Budapest

**Fig. 5:**
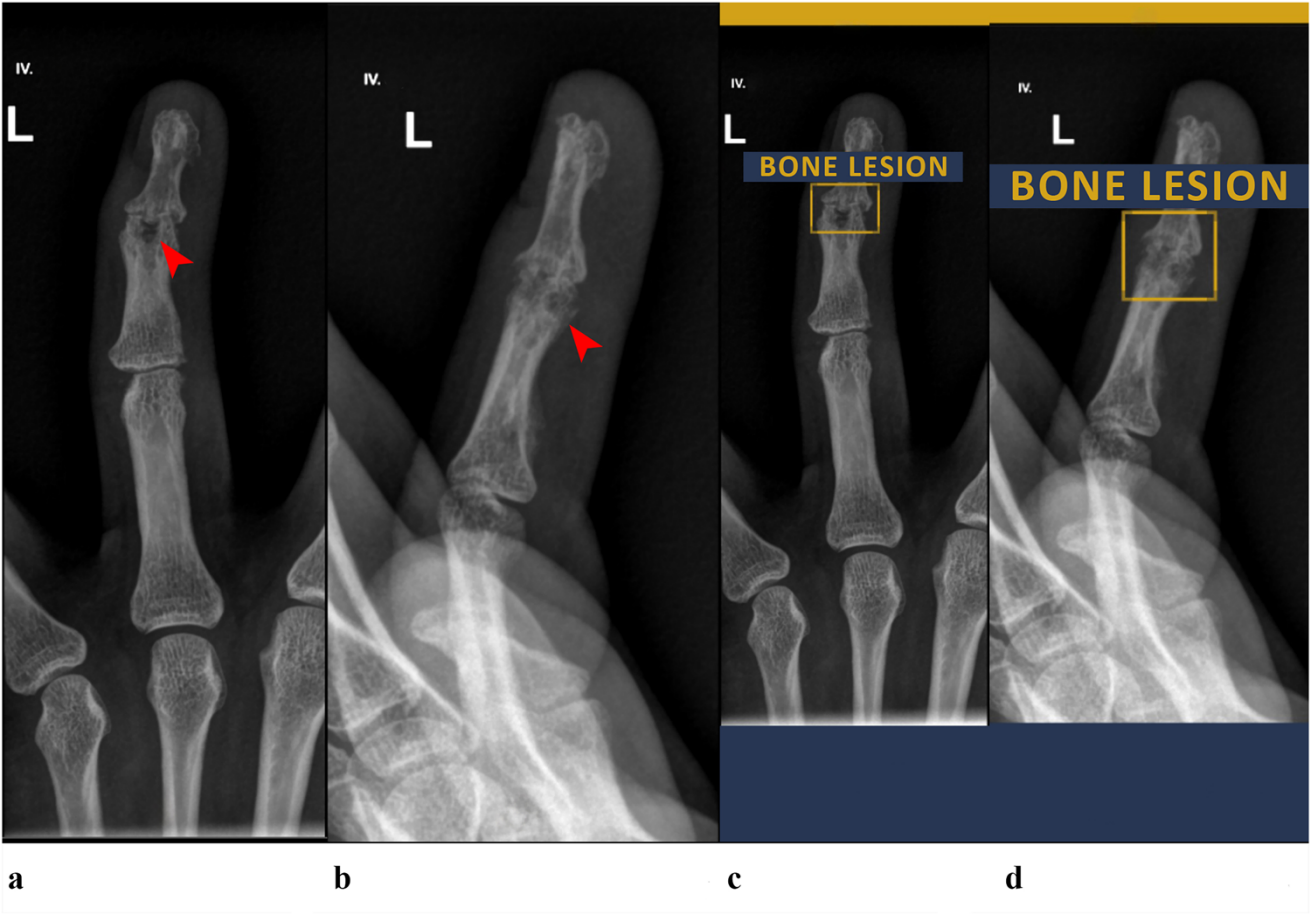
Detection of chronic bone lesions in post-traumatic osteomyelitis. A 46-year-old female patient presented with a known history of osteomyelitis in the fourth finger of the left hand. The patient previously underwent percutaneous wire fixation according to the Ishiguro method following a comminuted fracture of the proximal phalanx. Current imaging revealed a persistent lytic lesion involving the distal and middle phalanges, consistent with chronic bone infection. **a** Anteroposterior view showing severe bone destruction in the distal phalanx and partial involvement of the middle phalanx (red arrow). **b** Lateral view further confirming cortical thinning and the lytic nature of the lesion (red arrow). **c**, **d** AI analysis correctly identifies the area of pathological change, labeling it as a “bone lesion” and localizing the affected segments with high precision. Expert adjudication confirmed that the AI detection aligned with the final clinical diagnosis, demonstrating the system’s capability to recognize non-fracture bone pathologies and support longitudinal case management. AI, Artificial intelligence*.*Source: Medical Imaging Centre, Semmelweis University, Budapest

Discrepancies between AI and radiologist interpretations were identified in 326 cases and were most frequently associated with challenging radiographic features, including postoperative change and anatomical variants such as accessory ossicles (Fig. 2). The AI demonstrated a limited distinction between neighboring fractures, which can be explained by its two-stage object detection architecture, which lacks segmentation capability. This technical limitation was illustrated in Fig. 3, where two anatomically distinct fracture lines were counted as a single lesion. Finally, Fig. 4 demonstrates an example of joint diagnostic blind spots as a unique category of missed cases, emphasizing the ongoing need for human oversight, continual AI model refinement, and expanded validation across a broader spectrum of clinical presentations. These discrepancies (Fig. 5) were reviewed and adjudicated by an expert radiologist to refine the ground truth labeling.

## Discussion

This retrospective study evaluated the diagnostic performance of an AI-based fracture detection system across two distinct clinical settings: standalone AI *versus* radiologist interpretation, and AI-assisted radiologist interpretation *versus* AI alone. In the first cohort, the AI demonstrated expert-level performance at both the fracture and patient levels. Notably, the AI detected fractures in 63 cases that the radiologist missed, while the radiologist identified 71 fractures not flagged by AI, underscoring complementary strengths.

When AI support was made available to radiologists in routine workflow, performance patterns shifted. Radiologists assisted by AI achieved a higher positive predictive value (PPV) than the AI alone, suggesting a more precise identification of true-positive fracture cases. This likely reflects a cautious interpretation strategy in which the radiologist confirmed a fracture only when confident, possibly overriding ambiguous AI suggestions. While this approach improved diagnostic precision, it was associated with reduced sensitivity, indicating that subtle fractures flagged by the AI may have been overlooked.

Patient-level evaluation showed that the discrepancy rate slightly declined in the AI-assisted cohort (14.4% *versus* 15.6%), indicating potential for AI to reduce interpretation variability and enhance diagnostic consistency, even in settings where its output is used selectively rather than systematically. Taken together, these findings suggest that the AI system is capable of functioning as a reliable diagnostic tool in musculoskeletal trauma imaging. Its standalone performance not only approximated that of experienced radiologists but, in some cases, outperformed AI-assisted human interpretation.

Recent real-world studies have evaluated the clinical impact of AI-assisted fracture detection using controlled reader designs or mandatory AI exposure. The prospective study by Prucker et al demonstrated reduced reading time and increased diagnostic confidence with AI assistance, but no significant improvement in fracture detection performance when compared to unaided radiologists [19]. The “AI safety net” strategy proposed by Loeffen et al [23] suggested implementing AI subsequently as an independent second reader after the radiologists’ first interpretation to mitigate missed diagnoses by flagging discrepancies or overlooked findings. By functioning as a backstop rather than a primary decision-maker, AI significantly reduces false negatives, particularly missed fractures, while allowing radiologists to maintain clinical autonomy. The trade-off is an increase in false positives, but in trauma imaging, where the cost of a missed fracture can be substantial, this compromise is often regarded as acceptable.

The systematic review by Kwee et al reported heterogeneous effects of BoneView on sensitivity and specificity across readers and study designs, highlighting substantial methodological variability and potential bias [21]. The study on spinal compression fractures by Chen et al (2022) highlighted the potential of AI in radiography, as well as its technological constraints. Their findings of projection-dependent performance and modest accuracy support the idea that fracture morphologies and anatomical locations may affect AI strength, necessitating stratified investigations in subsequent research [24]. Such stratification could yield more granular insights into AI strengths and weaknesses. To support these efforts and address projection-dependent limitations, integrating deep learning networks capable of automatically classifying radiographic projections and body sides could serve as a vital preprocessing step. As recently demonstrated by Fink et al, such presorting algorithms not only optimize general radiologic workflow but can also minimize misclassifications in downstream AI systems, ensuring that fracture detection models receive standardized and accurately labeled inputs [25].

Several studies have demonstrated high diagnostic performance of AI tools in fracture detection, often reporting sensitivities above 90% in controlled settings. For example, Jacques et al (2023) found that a commercially available AI algorithm significantly improved radiologists’ sensitivity for wrist and hand fracture detection when compared to CT-based ground truth, without reducing specificity [26]. A systematic meta-analysis by Husarek et al [2] similarly showed that stand-alone AI models, particularly including BoneView, achieved a pooled sensitivity of 91% and a specificity of 89% when detecting fractures on conventional radiographs [2]. Our findings align well with this literature. Our investigation, however, uniquely evaluated AI performance in the daily emergency radiology workflow, where AI usage was discretionary rather than protocolized. This distinction is critical, as it reflects real-world clinical behavior rather than experimental exposure to AI output. In our real-world cohort with optional AI usage, standalone AI achieved a sensitivity of 93.5% fracture-wise and 95.6% patient-wise, while still displaying a high specificity of 94.6% and 97.1%, closely matching the AI-assisted radiologist. Notable strengths of this study include its large sample size of 2,317 trauma-related radiographic examinations, covering a broad anatomical distribution and reflecting the complexity of routine traumatology imaging. In addition to acute trauma detection, our dataset also included complex and chronic cases, such as osteomyelitis-associated lytic lesions. These were accurately flagged by the AI system, suggesting an extended diagnostic utility that may support longitudinal care beyond the scope of initial trauma triage. By evaluating performance on both a fracture-wise and patient-wise level, the analysis captures subtle diagnostic differences that may otherwise be overlooked in simpler case-based metrics. Thus, our reported results underscore the promise of AI as a second reader in trauma radiography, supporting clinical decision-making without replacing human judgment.

This study has several limitations that should be considered when interpreting the findings. First, while the sample size was substantial, the research was conducted at a single center, which may limit the generalizability of results to other clinical environments or populations. Furthermore, our analysis did not stratify fracture detection performance by anatomical location, fracture morphology, degree of displacement, or joint involvement. The study was limited to x-rays, not investigating cross-sectional imaging examinations. The AI use in the second cohort was discretionary and was not systematically recorded, resulting in inconsistent use among radiologists and introducing potential reader bias and confirmation bias. The decision to classify all “suspicious” findings as positive likely increased sensitivity, but may have influenced the overall diagnostic behavior and introduced interpretation ambiguity. Importantly, a technical limitation of the AI model used in this study is that its backbone is a two-stage object detection architecture, which lacks segmentation capability, resulting in a lack of distinction between two closely attached fractures. This aligns with our previous observations that detection and localization are distinct tasks, and that AI systems optimized for object-level recognition may struggle with fine-grained segmentation in clustered fracture scenarios [27].

Future research should clarify under what conditions AI support yields consistent diagnostic benefit and how integration protocols might be optimized. Prospective studies with controlled implementation of AI assistance, such as requiring radiologists to acknowledge or respond to AI suggestions, could provide deeper insights into human-AI interaction and its impact on diagnostic accuracy. Multi-center trials across diverse healthcare settings could assess generalizability and robustness, especially in hospitals with limited subspecialty coverage. Furthermore, anatomical-region-wise subanalyses are needed to identify areas where AI adds the most value. To extend our observations, future work could also evaluate the impact of AI integration on time-to-diagnosis, clinical workflow efficiency, and downstream outcomes such as treatment delays or repeat imaging rates. Finally, non-inferiority analyses and long-term deployment monitoring will be essential for establishing AI as a safe and effective diagnostic adjunct in musculoskeletal trauma imaging.

In conclusion, this study demonstrates that a standalone AI system achieves high diagnostic performance in musculoskeletal trauma imaging, often statistically outperforming AI-assisted radiologists in both sensitivity and accuracy. AI-assisted reporting was associated with reduced discrepancy rates and higher PPVs, suggesting it can enhance diagnostic consistency and precision. Ultimately, while the AI system proves to be a reliable and promising diagnostic tool, these findings indicate that maximizing its clinical value requires standardized integration protocols to overcome confirmation biases and ensure effective human-AI collaboration.

## Data Availability

If needed, raw data, datasheets, and statistical analysis of the abovementioned work could be provided.

## Abbreviations

AI: Artificial intelligence
ED: Emergency departments
NPV: Negative predictive value
PACS: Picture archiving and communication system
PPV: Positive predictive value
